# Homologous–heterogeneous structure control and intelligent adsorption effect of a polycationic gel for super-efficient purification of dyeing wastewater

**DOI:** 10.1039/c9ra00690g

**Published:** 2019-03-25

**Authors:** Chunli Song, Hongyan Li, Yikai Yu

**Affiliations:** College of Chemistry and Chemical Engineering, Jiangxi Normal University Ziyang Road 99 Nanchang 330022 China yuyikai1980@163.com; Key Laboratory of Chemical Biology of Jiangxi Province Ziyang Road 99 Nanchang 330022 China

## Abstract

A homologous–heterogeneous polycationic gel (HPCG) system was constructed by a waste-free synthesis process, to be used as a super-efficient adsorbent material for purifying dyeing wastewater. It is the first discovery of a new intelligent adsorption effect occurring in HPCG adsorption by detecting the homologous–heterogeneous structure transformations in HPCG adsorption using optical microscopy, scanning electron microscopy, X-ray diffraction, and X-ray photoelectron spectroscopy analysis technologies. The adsorption capacities of HPCG products were 532.55–605.45 times higher than that of the widely-used activated carbon, thereby being the greatest improvement of the adsorption ability for HPCG *versus* this existing adsorbent. Meanwhile, the adsorption capacities of HPCG were also improved by 3.67–46.05 times compared to that of all the similar polycationic cotton adsorbents reported in our previous serial works, demonstrating more efficient purification of dyeing wastewater than we could do before. In addition, through studying the adsorption models, it was further discovered that HPCG adsorption followed the new two-segment adsorption process, *i.e.* including a speed control segment and an acceleration segment, also confirming the existence of the intelligent adsorption effect for HPCG adsorption.

## Abstract 2

A homologous–heterogeneous polycationic gel (HPCG) system was constructed by a waste-free synthesis process, to be used as a super-efficient adsorbent material for purifying dyeing wastewater.

## Graphical abstract

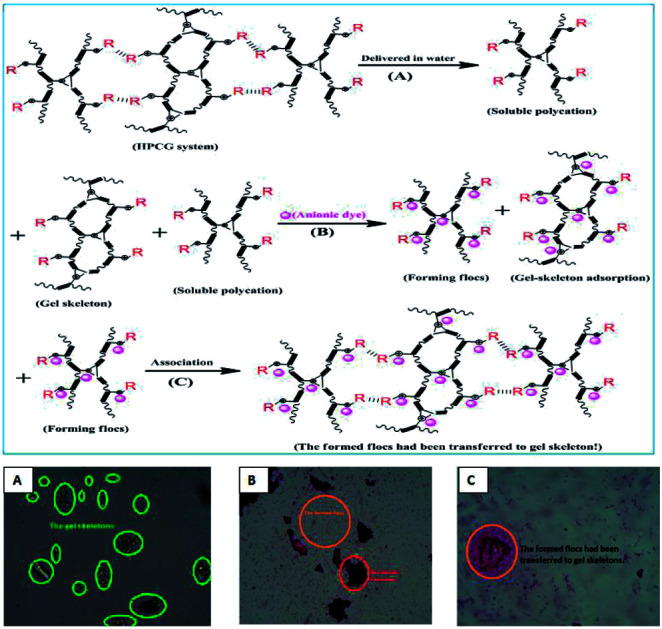

## 1. Introduction

The disorderly emissions of dyeing wastewater have caused serious environmental pollution and destroyed global sustainable development.^1–5^ The coloured water-soluble dyes that are the main components in dyeing wastewater are especially difficult to remove because they are highly dispersed in the water phase with stable molecular forms. Thus, the removal of the coloured water-soluble dyes should be regarded as one of the most critical things in the whole purification process of dyeing wastewater.^6–10^ Adsorption has become one of the most widely-used and convenient methods for water treatment, and the key is the suitable selection of adsorbent materials. The traditional adsorbent materials for purification of dyeing wastewater are mainly activated carbon adsorbent, clay mineral (or solid waste) adsorbent, and natural product adsorbent. Activated carbon and clay mineral were two of the first adsorbents used.^11–15^ Due to the porosity, network, and large specific surface structures, they could produce a certain level of Van Edward forces for the deep treatment (*e.g.*, tertiary treatment) of small-sized pollutants in dyeing wastewater, but the direct adsorption of large-sized dyes is very weak, and therefore should be combined with some oxidising substances (*e.g.*, O_3_, ClO_2_, H_2_O_2_, CuO, Fe_2_O_3_, V_2_O_5_, and Fenton reagent) to decompose them, after which they can be adsorbed. However, the solid wastes of the activated carbon and clay mineral (or solid waste) are slow to self-decompose and therefore still exist in the environment for a long time, which are is likely to cause a secondary pollution.^16–20^ In recent years, researchers have focused on the environmentally friendly and low cost natural product adsorbents. Nevertheless, the adsorptions of these traditional adsorbents mainly rely on the intermolecular forces, which are relatively weaker than normal chemical bonds, suggesting that the adsorption forces of traditional adsorbents could be further strengthened.

We observed that several polycationic skeletons [*e.g.*, polydimethyldiallylammonium chloride (PDMDAAC)] contained the high densities of cationic adsorption points which could produce efficient electrostatic attractions (*i.e.* ionic bonds) towards the coloured anionic dyes,^20–30^ when they were incorporated into cotton skeletons, to obtain a series of new polycationic cotton adsorbents reported in our previous serial works.^31–33^ It was observed that the adsorption capacities of all the polycationic cotton adsorbents were improved 17.4–145.3 times that of the widely-used activated carbon due to the formation of the strong electrostatic adsorption interactions (*i.e.* ionic bonds) between these polycationic cottons and the coloured anionic dyes. In the most recent work of the crosslinking polycationic poly (triallylmethylammonium chloride) cotton (PT-cotton), we first discovered a new sign that the gel adsorption effect of the crosslinking polycationic structures on PT-cotton surfaces played an advantageous role in improving the adsorption capacities.^33^ This new sign further lead us to investigate whether the crosslinking polycationic gel-structures themselves would have the improved adsorption abilities of a new type of adsorbent material to purify dyeing wastewater more efficiently than we could do before.

**Scheme 1:**
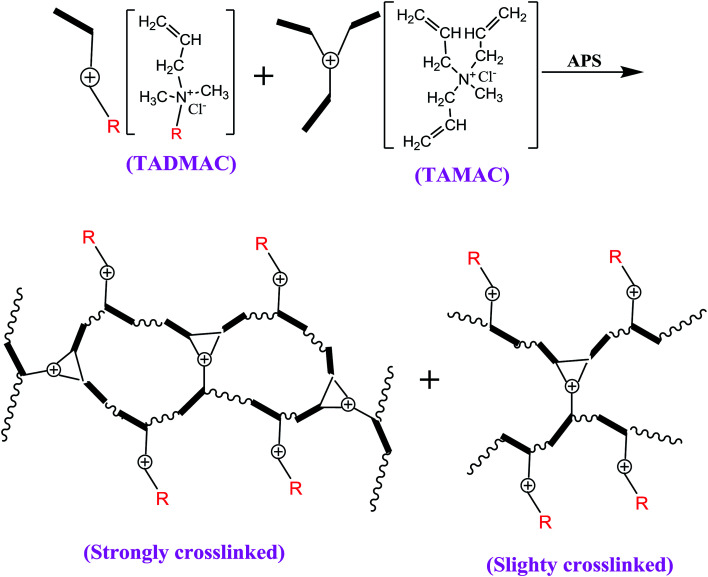
Design and construction of homologous–heterogeneous HPCG system. (*) R: tetradecyl group.

In this work, we specially designed a new crosslinking homologous–heterogeneous polycationic gel (HPCG) as a new type of adsorbent system for purifying dyeing wastewater. It can be synthesized by a crosslinking copolymerization of one crosslinking cationic monomer (*i.e.* triallylmethylammonium chloride, TAMAC) and another cationic monomer containing a long-chain alkyl group (*i.e.* tetradecylallyldimethyl ammonium chloride, TADMAC). It is the first study that shows that the heterogeneous structures derived from this same crosslinking copolymerization system (*i.e.* the homologous system) could play roles in improving the adsorption abilities of obtained HPCG systems. In the crosslinking copolymerization system, on the one hand, TAMAC and TADMAC monomers could be strongly crosslinked to form insoluble network polycations, which would be as solid forms of gel skeletons to adsorb and fix the anionic dyes in their application. On the other hand, from the perspective of reaction probability, there was also the probability for TAMAC and TADMAC monomers to be slightly crosslinked to form the soluble plane-like polycations, which would be soluble and delivered in water as the liquid forms of the anionic-dye scavengers to efficiently catch the anionic dyes in water. This meant that the obtained HPCG adsorbent system had the homologous–heterogeneous structures with both the insoluble network and the soluble plane-like polycations (Scheme 1). In the application, the adsorption capacities of HPCG products with the homologous–heterogeneous structures were 532.55–605.45 times higher than that of the widely-used activated carbon, being the greatest improvement of the adsorption ability of HPCG *versus* this existing adsorbent. Meanwhile, the adsorption capacities of HPCG were also improved by 3.67–46.05 times that of all the similar polycationic cotton adsorbents reported in our previous serial works, thus displaying more efficient purification of dyeing wastewater than we could do before. Moreover, it was the first discovery of a new intelligent adsorption effect occurring in HPCG adsorption, determined by comparing the homologous–heterogeneous structure transformations in HPCG adsorption using optical microscopy, scanning electron microscopy, X-ray diffraction, and X-ray photoelectron spectroscopy analysis technologies.

In addition, in the designed synthesis process, almost all of the raw materials could be changed into the useful components of HPCG adsorbent systems, and be entirely used up without any treatments and waste, whereas in many synthesis processes, the unreacted materials or by-products are usually treated as waste, resulting in high preparation costs and environmental pollution. Thus, this designed synthesis process is relatively economical and green.

## 2. Experimental

### 2.1 Materials

Tetradecylallyldimethyl ammonium chloride (TADMAC) was prepared by a quaternisation reaction of tetradecyldimethylamine and allyl chloride at 55–65 °C in C_2_H_5_OH solvent for 24 h according to the method in our previous study.^32^ Ammonium persulfate (APS) was purchased from Yixing Tianpeng Fine Chemical Co., Ltd (China). Reactive Scarlet 3BS (industrial purity) was purchased from Jiangsu Nantong Si'ente Chemical Co., Ltd (China).

### 2.2 Method

#### 2.2.1 TAMAC synthesis

Triallylmethylammonium chloride (TAMAC) was prepared by a quaternisation reaction of diallylmethylamine and allylchloride using dry acetone as the solvent at 35 °C for 48 h according to another of our previous studies.^33^

#### 2.2.2 Waste-free synthesis of homologous–heterogeneous HPCG products

In a 50 mL round-bottomed flask, 5.5 g of TAMAC, 5.5 g of TADMAC, 0.55 g of ammonium persulfate (APS, initiator), and 9.0 mL of deionised water were mixed evenly to create the reaction solution. The reaction solution was warmed at 50 °C for 3.0 h and was then further cured by raising the temperature to 75 °C with 0.5 g of the mixture initiator (NH_4_)_2_S_2_O_8_–NaHSO_3_ for 2 h, to obtain a model product of HPCG with the molar ratio of TAMAC and TADMAC units being 50/50. *Via* the same synthesis conditions mentioned above, a series of HPCG products could be systemically synthesised by varying the molar ratios of TAMAC and TADMAC monomers from 100/0 to 10/90. The obtained HPCG products could be directly used without any treatments and waste to purify dyeing wastewater.

### 2.3 Adsorption studies of HPCG products

Firstly, the adsorption experiment of one HPCG adsorbent with the molar ratio of TAMAC and TADMAC units being 60/40 toward a large-sized anionic dye (*i.e.* Reactive Scarlet 3BS) was selected as the model experiment. In 100 mL of a 100 mg L^−1^ dye solution of Reactive Scarlet 3BS, several dosages (0.007–0.03 g) of HPCG samples were added. The adsorption experiments of the selected HPCGs toward the selected dyes were conducted at 30 °C for 100 h, until the dye concentration in the solution no longer changed, thereby attaining adsorption equilibrium. The concentration of the dye residue in the solution could be measured by a spectrophotometer, allowing us to obtain the dye removal percentage (*R*%, the amount of dye removed as a percentage of the initial amount of dye) at different dosages in order to evaluate the adsorption abilities of HPCG products.

Subsequently, based on the dye removal percentages at different dosages, the equilibrium dye concentration in the solution (*C*_e_) and the equilibrium adsorption capacity (*q*_e_) of HPCG could be further calculated, which could be selected to fit the Langmuir and Freundlich equations for evaluating the adsorption isotherm of the variable relationships between *q*_e_ and *C*_e_.

Langmuir equation: 1
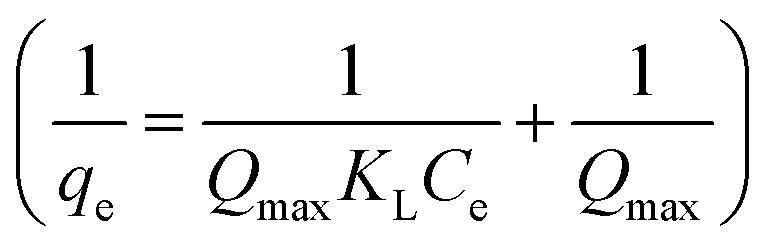


Freundlich equation2
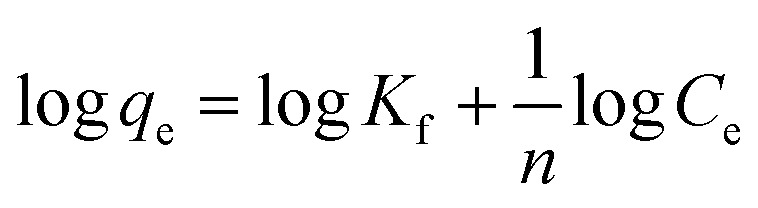


*K*_L_ is the characteristic constant of the Langmuir equation, *Q*_max_ is the maximal adsorption capacity of the Langmuir equation, and *K*_f_ and *n* are the characteristic constants of the Freundlich equation representing the adsorption capacity.

Moreover, the adsorption ability of HPCG at different times were investigated as follows: 0.016 g of HPCG samples were added to 100 mL of a 100 mg L^−1^ dye solution of Reactive Scarlet 3BS at 30 °C for 10 to 40 min with continuous stirring, then the dye adsorption in the solution at each time interval (*t*) was determined by a spectrophotometer, for calculating the dye removal percentage (*R*%) at time *t*. Based on the dye removal percentage (*R*%) at time *t*, the dye concentration in the solution (*C*_*t*_), and the amount of dye adsorption (*q*_*t*_) at time *t* could be further calculated. These were used to fit the typical adsorption kinetics equations (eqn (3): pseudo-first kinetics equation, eqn (4): pseudo-second kinetics equation, eqn (5): intraparticle diffusion equation, and eqn (6): particle diffusion equation).

Pseudo-first order kinetic model3
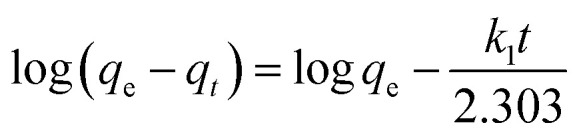


Pseudo-second order kinetic model4
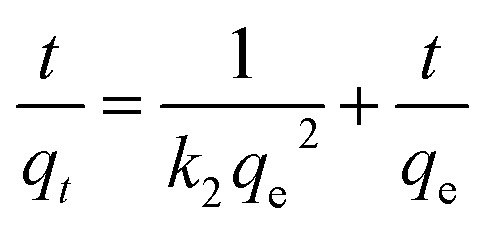


Intra-particle diffusion model

5 *q*_*t*_ = *k*_i_*t*^1/2^ + *x*_i_

Particle diffusion model6
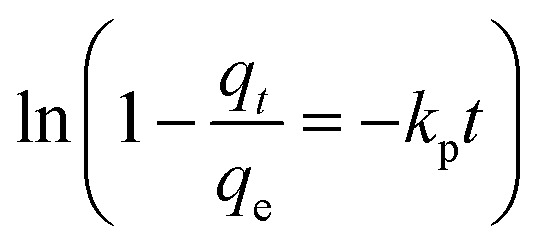


The slopes and intercepts of the liner plots of eqn (3)–(6) could give the adsorption rate constants corresponding to the pseudo-first order kinetics (*k*_1_), pseudo-second order kinetics (*k*_2_), intraparticle diffusion (*k*_i_), and particle diffusion equations (*k*_p_), respectively, all of which could be used to evaluate the adsorption kinetics behaviours of HPCG.

### 2.4 Measurement

Fourier transform infrared spectra (FT-IR) were recorded on a Nicolet FT-IR (510 P, USA) spectrophotometer using disk method within the wavelength range of 4000–400 cm^−1^.

X-ray photoelectron spectroscopy (XPS) measurements were carried out by a Thermo VG multilab 2000 spectrometer with an Al Kα X-ray source.

X-ray diffraction (XRD) analysis, was conducted with a Rigaku D/MAX-IIA X-ray diffractometer, using CuKα radiation at 30 kV and 20 mA, with the diffractograms recorded at room temperature over the range 2*θ* = 10 to 90°.

Optical microscope photos for HPCG adsorption in water were observed by a 35 TV optical microscope instrument with a computer camera.

Scanning electron microscopy (SEM) analysis was carried out using a JSM-5610 SEM instrument.

The maximum absorbance wavelength of the selected dye solution (588 nm, Reactive Scarlet 3 BS) was determined with a U-3310 UV-Vis Spectra. At the maximum absorbance wavelength of 588 nm, the dye absorbance in the untreated solution (*A*_0_) and in the PA-cotton treated solution (*A*_t_) could be measured by a Shanghai 721 spectrophotometer. The dye removal percentage *R*% could be calculated from the percentage absorbance using the following equation (eqn (7)):7
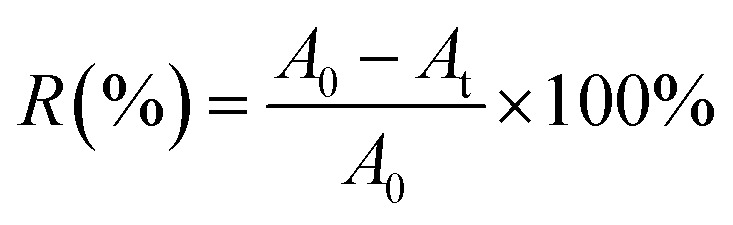


The monomer conversions (*C*%) could be approximately assessed by measuring the moles of the residual double bonds in the residual monomers^20^ and were calculated according to the following equation (eqn (8)).8
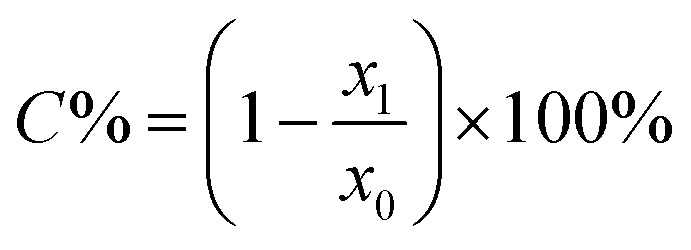
where *x*_1_ was the moles of residual bonds and *x*_0_ was the total moles of double bonds.

## 3. Results and discussion

### 3.1 Optimizing the waste-free synthesis of HPCG products

First, we selected one model synthesis of HPCG adsorbent with the molar ratios of TAMAC and TADMAC monomers being 50/50 as the optimum example. Subsequently, an orthogonal experiment with four factors [initial reaction temperature (factor *A*), monomer concentration (factor *B*), APS initiator amount (factor *C*), and reaction time (factor *D*)] and three levels was designed to investigate the effect of different conditions on the optimal synthesis of HPCG adsorbents. The optimal experiments were conducted according to an L_9_ (3)^4^ matrix. The monomer conversion percentages under each condition were used to evaluate the result effects. The results were shown in Table 1. When the monomer conversion percentage reached its highest level, the optimum synthesis conditions of HPCG adsorbent could be confirmed: the initial reaction temperature was 50 °C, the monomer concentration was 60% (w/w), the APS initiator amount was 5% (w/w), and the reaction time was 3.0 h. Under the optimal conditions, the monomer conversion percentage was 91.9%. When most of the synthesis reaction of TAMAC and TADMAC monomers was accomplished under the optimum conditions, the reaction solution could be further cured by raising the reaction temperature to 75 °C with a more active initiator of (NH_4_)_2_S_2_O_8_–NaHSO_3_ for 2 h, so that the monomer conversion percentage in the reaction solution could be stably increased to 95.8%. *Via* the same optimal synthesis conditions, a series of HPCG products were systemically synthesised by varying molar ratios of TAMAC and TADMAC monomers from 100/0 to 10/90, and the monomer conversion percentages in the reaction solutions were above 93.2% (Table 2), indicating that the designed synthesis process was appropriate to obtain the expected HPCG products.

**Table 1:** The L_9_ (3)^4^ for the synthesis of HPCG products^a^

No.	*A* (°C)	*B* (w/w, %)	*C* (w/w, %)	*D* (h)	Monomer conversion (%)
1	45	50	5	2	73.6
2	45	55	10	3	77.1
3	45	60	15	4	81.8
4	50	50	10	4	86.4
5	50	55	15	2	90.5
6	50	60	5	3	91.9
7	55	50	15	3	88.3
8	55	55	5	4	81.7
9	55	60	10	2	92.2

a *A*: initial polymerization temperature, *B*: monomer concentration, *C*: initiator amount, *D*: polymerization time.

**Table 2:** Properties of serial HPCG products

No.	*n*_(TAMAC)_/*n*_(TADMAC)_	Monomer conversion (%)
1	100/0	97.9
2	80/20	97.4
3	70/30	96.8
4	60/40	96.2
5	50/50	95.8
6	40/60	95.2
7	30/70	94.7
8	20/80	94.0
9	10/90	93.2

In addition, the obtained HPCG products could be directly used without any treatments or waste, showing that designed synthesis process is more economical and green than the traditional synthesis processes that the unreacted materials or by-products are usually treated as waste.

### 3.2 Analysis of the homologous–heterogeneous structures of HPCG products

**Fig. 1:**
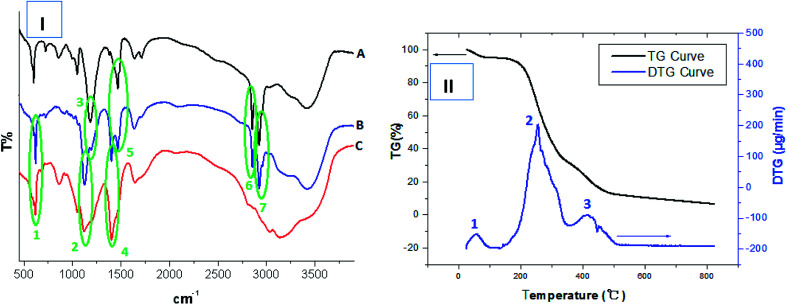
(I) Comparing FT-IR analysis of TADMAC self-polymer (curve “A”), HPCG product (curve “B”) and TAMAC self-polymer (curve “C”). (II) TG analysis of obtained HPCG product.

The structures of the obtained HPCG products were analysed by Fourier transform infrared spectrum (FT-IR) and thermogravimetric (TG) analysis. The results are shown in Fig. 1(I and II).

The FT-IR spectrum of the selected HPCG product (with the molar ratios of TAMAC and TADMAC units being 50/50) [curve “B” in Fig. 1(I)] showed the same absorption as the FT-IR spectrum of the self-polymer of TAMAC monomers [curve “C” in Fig. 1(I)] at 614 cm^−1^ (peak 1), 1116 cm^−1^ (peak 2), and 1399 cm^−1^ (peak 4), respectively. It also showed the same absorption as the FT-IR spectrum of the self-polymer of TADMAC monomers [curve “A” in Fig. 1(I)] at 1178 cm^−1^ (peak 3), 1461 cm^−1^ (peak 5), 2847 cm^−1^ (peak 6), and 2917 cm^−1^ (peak 6), respectively. This indicated that the HPCG product was obtained from the co-polymerization of TAMAC and TADMAC monomers as expected.

Moreover, the TG analysis technology was used to further analyse the structures of the HPCG product, and simultaneously showed the thermogravimetric analysis curve (TG curve) and the derivative thermogravimetric curve (DTG curve) [Fig. 1(II)]. In this case, the TG curve recorded the general mass loss trend of the HPCG sample with the increase of the test temperatures, and the DTG curve recorded the detailed mass loss process of the HPCG sample. From the perspective of the DTG curve, besides a water mass loss process at 21.08–100.33 °C (peak 1), there were two main mass loss processes of the HPCG sample occurring at 136.30–351.28 °C (peak 2) and 366.75–488.52 °C (peak 3), respectively, which could possibly be derived from the decomposition of the two homologous–heterogeneous components (*i.e.* the soluble plane-like polycations and the insoluble network gel-skeletons) in the HPCG system. Thus, the TG analysis results confirmed that the obtained HPCG products should have two homologous–heterogeneous structures with both the insoluble network gel-skeletons and the soluble plane-like polycations.

### 3.3 The evolution of adsorption abilities of HPCG products to realise the super-efficient purification of dyeing wastewater

**Fig. 2:**
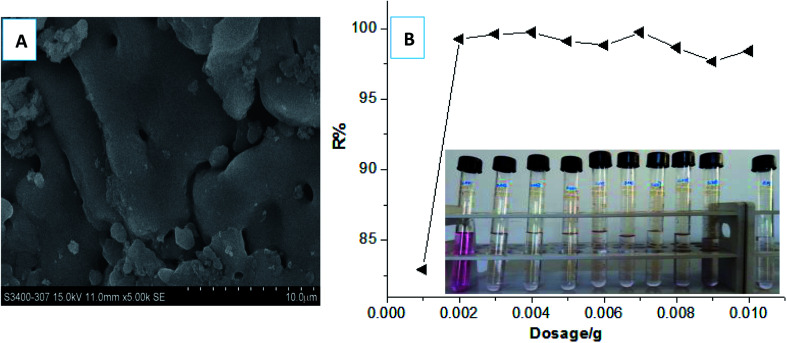
SEM analysis of morphological constructions of TAMAC homo-polymer gels (A). Comparing the adsorption abilities of the crosslinking TAMAC homo-polymer gels at different dosages (B)*. (*) The original adsorption effect at each condition could be correspondingly tracked in photos. Test conditions: 0.001–0.01 g of the crosslinking homo-polymer gels were used to adsorb 10 mL of 100 mg L^−1^ dye solution of Reactive Scarlet 3BS (a large-sized dye) for 100 h at 30 °C with magnetic stirring, and then filtered to detect the adsorption results.

In our most recent work,^33^ we first discovered a gel adsorption effect of the crosslinking polycationic structures derived from the homo-polymerization of the crosslinking cationic monomers (TAMAC) on PT-cotton surface, to play a more advantageous role in improving the adsorption capacities. This new sign interested us to further investigate whether the crosslinking polycationic gel-structures themselves would have better adsorption abilities as a new type of adsorbent materials. Firstly, we specially synthesised the crosslinking homo-polymers of the crosslinking cationic TAMAC monomers used as new crosslinking polycationic gel adsorbents and investigated the adsorption abilities. The results were shown in Fig. 2. The results showed, the construction of the TAMAC homo-polymer gel was a porous structure [Fig. 2(A)], which would be helpful by swelling in water and provide more spaces to absorb and accommodate the anionic dyes. A satisfactory dye removal percentage of 99.26% could be achieved when a low dosage of 0.002 g of the crosslinking TAMAC homo-polymer gels were used to adsorb 10 mL of a 100 mg L^−1^ dye solution of Reactive Scarlet 3BS (a large-sized dye) for 100 h at 30 °C [Fig. 2(B)]. The maximal adsorption capacity (*Q*_max_) could be calculated by the average adsorption values at the saturated adsorption states with dosages of 0.001–0.002 g. The *Q*_max_ of the crosslinking TAMAC homo-polymer gels was calculated as 662.9 mg g^−1^, which was 2.45 times higher than that of the previous PT-cotton adsorbent, confirming the assumption that the crosslinking TAMAC homo-polymer gels themselves had the better adsorption abilities.

**Fig. 3:**
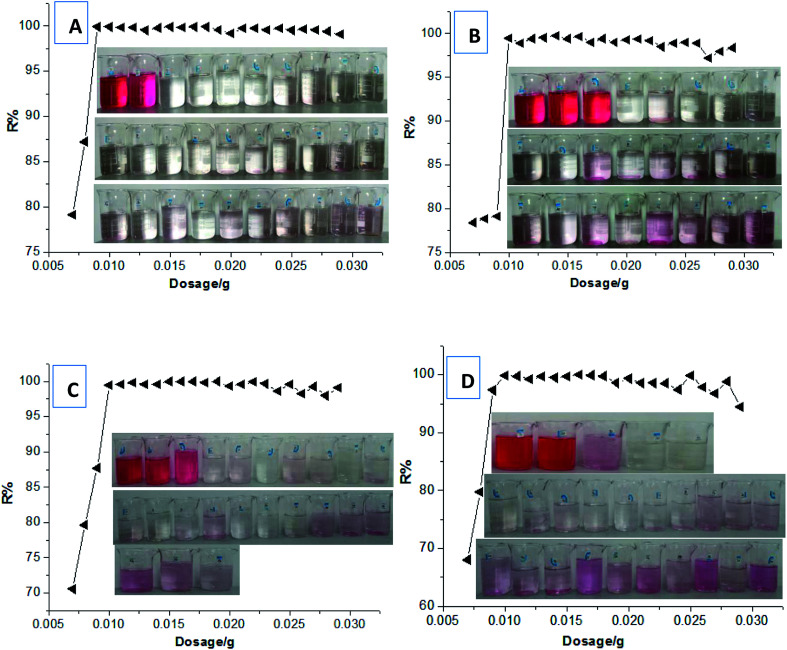
Comparing the adsorption abilities of different dosages of HPCG products with the molar ratios of TAMAC and TADMAC units being 80/20 (A), 70/30 (B), 60/40 (C), and 50/50 (D)*. (*) The original adsorption effect at each condition could be correspondingly tracked in photos. Test conditions: 0.007–0.029 g of HPCG products were added to adsorb 100 mL of 100 mg L^−1^ dye solution of Reactive Scarlet 3BS (a large-sized dye) for 100 h at 30 °C with magnetic stirring, and then filtered to detect the adsorption results.

Subsequently, we selected a functional cationic monomer (tetradecylallyldimethyl ammonium chloride, TADMAC) to be suitably co-polymerized with the crosslinking cationic TAMAC monomer, to construct the new HPCG adsorbent system. As designed, the obtained HPCG adsorbent system would have the homologous–heterogeneous structures with both the insoluble network gel-skeletons and the soluble plane-like polycations, and show intelligent adsorption behaviours to further improve their adsorption abilities. When the molar ratios of TAMAC and TADMAC monomers were controlled at the suitable ranges of 80/20–50/50, the obtained HPCG products with the molar ratios of TAMAC and TADMAC units being 80/20–50/50 could achieve the satisfactory dye removal percentages of 99.46–99.89% when their dosages were only 0.01 g to adsorb the 100 mL of a 100 mg L^−1^ dye solution of Reactive Scarlet 3BS. The detailed adsorption results were shown in Fig. 3. According to the average adsorption values at the saturated adsorption states with the dosages of 0.007–0.01 g in Fig. 3, the maximal adsorption capacities (*Q*_max_) of the HPCG products with the molar ratios of TAMAC and TADMAC units being 80/20, 70/30, 60/40, and 50/50 were calculated to be 1120.09 mg g^−1^, 1057.44 mg g^−1^, 1001.30 mg g^−1^, and 982.22 mg g^−1^, respectively, which were further improved 1.49–1.69 times compared to the previous crosslinking TAMAC homo-polymer gels, indicating that the construction of the new HPCG adsorbent system had been successful towards the super-efficient purification of dyeing wastewater as expected.

**Fig. 4:**
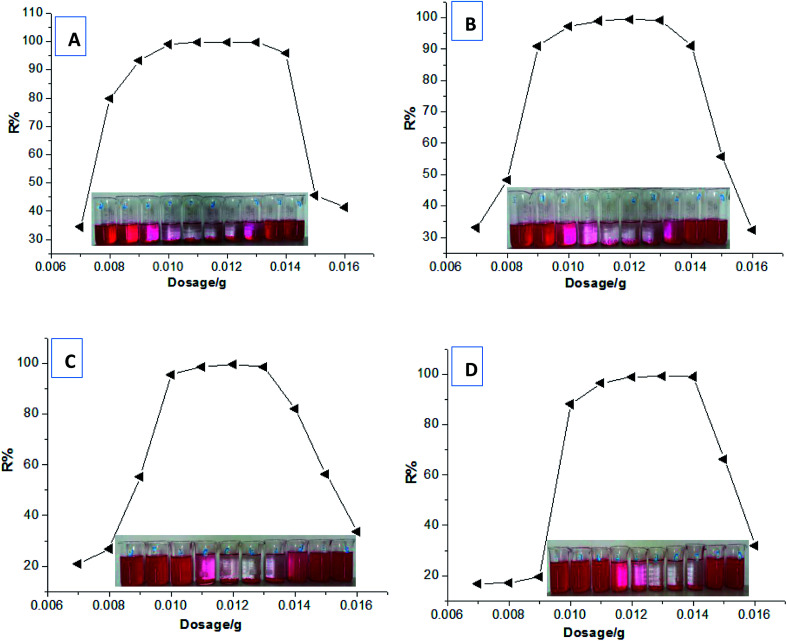
Comparing the adsorption abilities of different dosages of HPCG products with the molar ratios of TAMAC and TADMAC units being 40/60 (A), 30/70 (B), 20/80 (C), and 10/90 (D)*. (*) The original adsorption effect at each condition could be correspondingly tracked in photos. Test conditions: 0.007–0.029 g of HPCG products were used to adsorb 100 mL of 100 mg L^−1^ dye solution of Reactive Scarlet 3BS (a large-sized dye) for 100 h at 30 °C with magnetic stirring, and then filtered to detect the adsorption results.

In addition, when the molar ratios of TAMAC and TADMAC monomers were further decreased to 40/60–10/90, due to the decrease of crosslinking degrees in the molecular structures, the obtained polycations were slightly crosslinked and showed the plane-like structures, which were completely soluble in water and without the insoluble network skeleton structures. In this case, the soluble plane-like polycations could be independently used as the flocculants to form the insoluble flocs with the anionic dyes for purifying the dyeing wastewater (Fig. 4). However, good dye removal percentages (96.82–98.34%) could be attained only when the dosages were controlled at 100–150 mg L^−1^. When the dosages were more than 150 mg L^−1^ or less than 100 mg L^−1^, the percentages of dye removal became very poor (<90%). Thus, the good dye removal percentages of independent use of the soluble plane-like polycations only depended on precise control of the dosages, which would not be adaptable to the changeable water environment in real application. In further comparison to the HPCG systems mentioned above with the molar ratios of TAMAC and TADMAC units being 80/20–50/50, the application adaptability of only the soluble plane-like polycations (with the molar ratios of TAMAC and TADMAC units being 40/60–10/90) was poorer, such that there was not combination with the solid network gel skeletons.

Generally, it could be concluded from these results that those HPCG products with the suitable molar ratios of TAMAC and TADMAC units being 80/20–50/50 had the best adsorption abilities, and could be regarded as the optimum HPCG adsorbent products.

### 3.4 Comparing the adsorption superiority of HPCG products

Several typical and similar adsorbents were further selected to compare the superiority of HPCG's adsorption capacity, the test conditions of which were the same as those used for HPCG adsorption in this work. The results showed, the adsorption capacities of the optimum HPCG products were 532.55–605.45 times higher than that of the widely-used activated carbon, thereby being the greatest improvement of the adsorption ability for HPCG *versus* this existing adsorbent. Meanwhile, the adsorption capacities of HPCG were also improved 3.67–46.05 times that of all the similar polycationic cotton adsorbents (*i.e.* G-cotton, PF-cotton, LP-cotton, and PT-cotton) reported in our previous serial works^31–33^ (Table 3), to demonstrate more efficient purification of dyeing wastewater than we could before.

**Table 3:** Comparing the adsorption superiority of HPCG products to existing adsorbents under the same test conditions

Adsorbent	Adsorbate	*Q*_max_ (mg g^−1^)	Reference
Activated carbon	Reactive Scarlet 3BS	1.85	31
G-cotton	Reactive Scarlet 3BS	24.33	31
PF-cotton	Reactive Scarlet 3BS	37.10	31
LP-cotton	Reactive Scarlet 3BS	106.30	32
PT-cotton	Reactive Scarlet 3BS	268.82	33
HPCG^a^	Reactive Scarlet 3BS	985.22–1120.29	This work

a The optimum HPCG products with the suitable molar ratios of TAMAC and TADMAC units being 80/20–50/50 were selected as the comparison.

### 3.5 The homologous–heterogeneous structure transformations during HPCG adsorptions

**Fig. 5:**
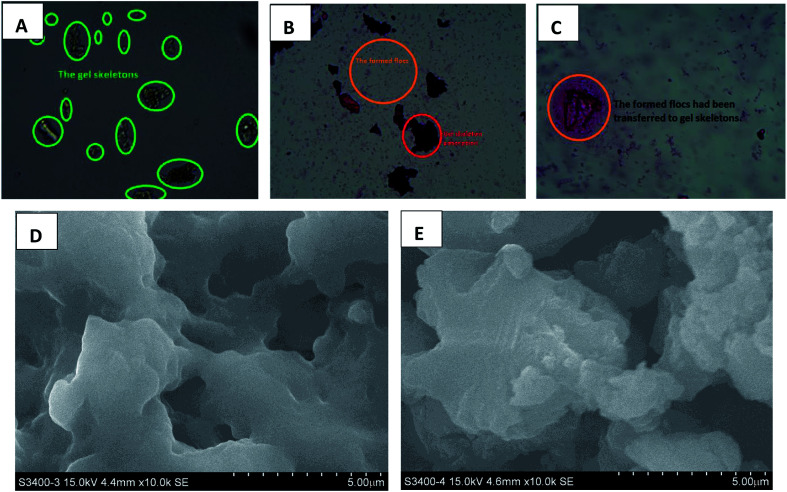
Comparing the optical microscopy analysis of the gel states of HPCG skeletons (A), the two-phase interaction states of homologous–heterogeneous polycations in HPCG system (B), and the states of transferring the flocs to HPCG skeletons (C). Comparing the SEM analysis of construction morphology before (D) and after (E) HPCG adsorption.

**Fig. 6:**
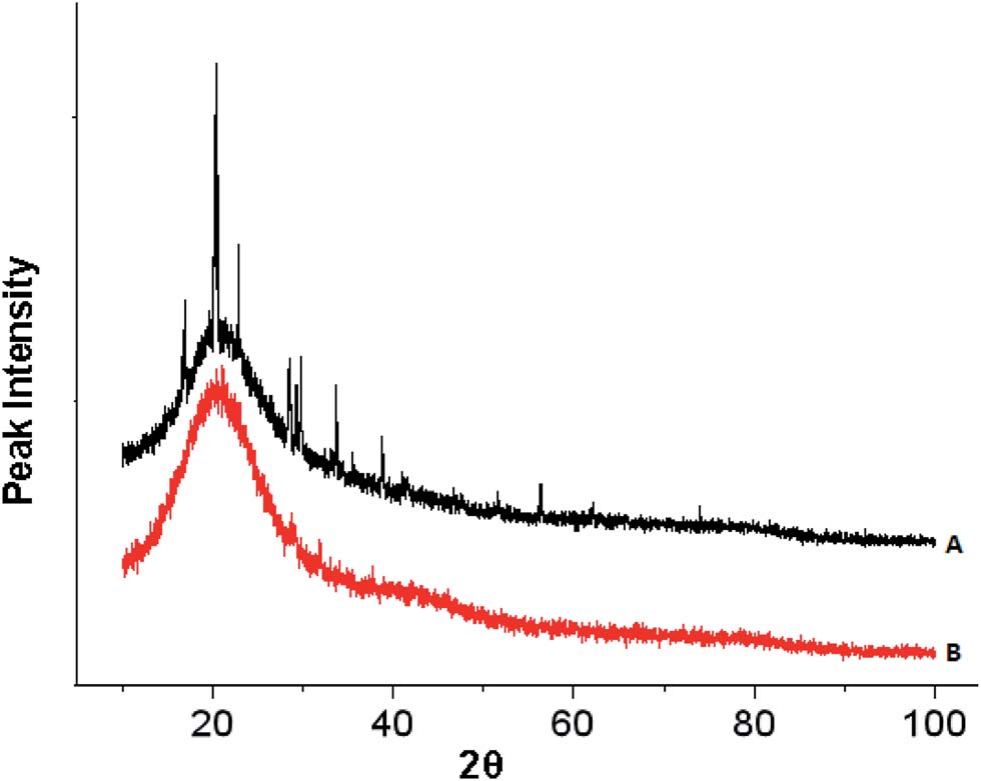
Comparing XRD analysis for detecting the crystalline structure transformations before (A) and after (B) HPCG adsorption.

**Fig. 7:**
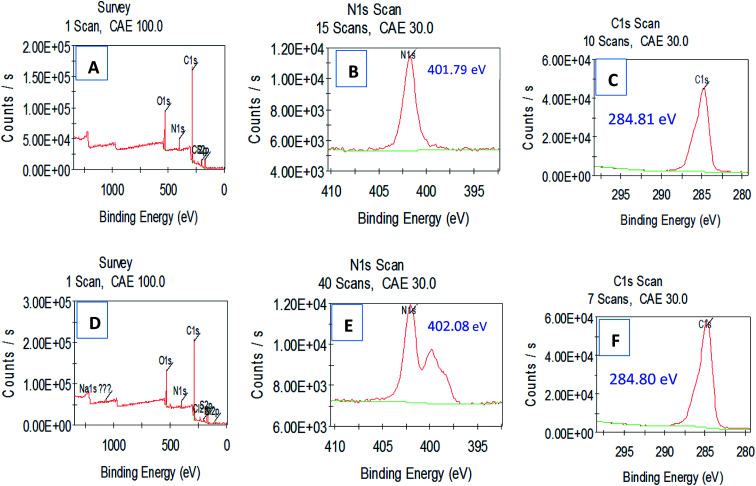
Comparing XPS analysis for detecting the interaction binding energy transformations before (A–C) and after (D–F) HPCG adsorption.

It was first studied that the homologous–heterogeneous structures of HPCG products play roles to improve the adsorption abilities, which were observed by optical microscopy, SEM, XRD, and XPS analysis technologies. The results were shown in Fig. 5–7, respectively.

**Scheme 2:**
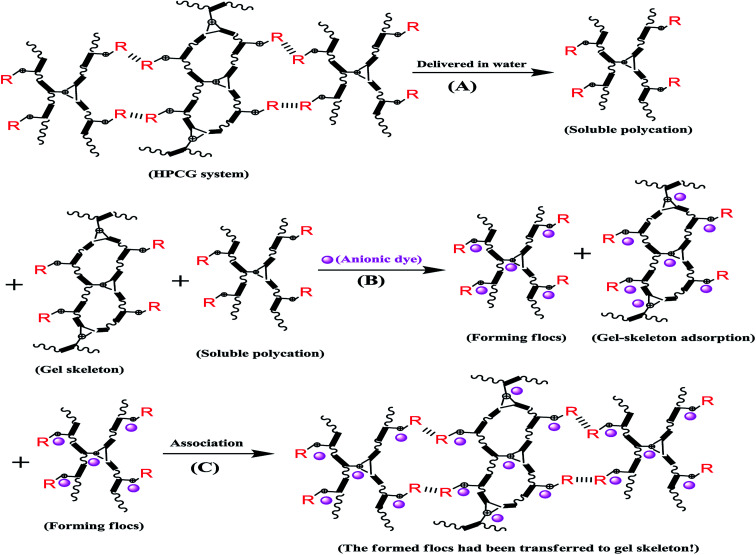
Drawing the intelligent adsorption processes of HPCG*. 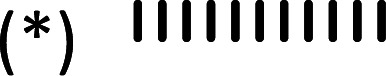: the association interactions between the alkyl groups (*i.e.* R: the tetradecyl groups). (A) The HPCG system delivered the soluble polycations as the anionic-dye scavengers in water and maintained the insoluble network polycations as the gel skeletons. (B) On the one hand, the gel skeletons in HPCG system could absorb and accommodate the anionic dyes. On the other hand, the soluble polycations could catch the anionic dyes in water and form the insoluble flocs with the anionic dyes. (C) The formed flocs were transferred to the solid gel skeletons to be further fixed due to the associations between the alkyl groups in the gel skeletons and those in the formed flocs.

An optical microscope with computer imaging (magnification of 200×) was adopted to monitor the adsorption behaviours of the HPCG product (with the molar ratios of TAMAC and TADMAC units being 50/50) in water, and observed that the obtained HPCG products could show the intelligent adsorption behaviours because of the homologous–heterogeneous structures [Fig. 5(A–C)]. On the one hand, the insoluble network polycations in the HPCG system could be swelling in water to form the transparent bodies as the solid forms of the gel skeletons [Fig. 5(A)], which could provide more spaces to absorb and accommodate the anionic dyes [see the field circled in red in Fig. 5(B)]. On the other hand, the soluble plane-like polycations in the HPCG system could be freely delivered into the water phase as the liquid forms of the anionic-dye scavengers, which could efficiently catch the anionic dyes, to form the insoluble flocs with the anionic dyes and separate them from water phase [see the emerging small coloured bodies in the yellow circled field in Fig. 5(B)], and then the formed flocs could be transferred to the solid gel skeletons to be further fixed due to the associations between the alkyl groups in the gel skeletons and those in the formed flocs [Fig. 5(C)]. The detailed intelligent adsorption processes of HPCG were drawn in Scheme 2. When the constructions before and after HPCG adsorptions were further observed with the greater magnification of 10000× by SEM [Fig. 5(D and E)], the results showed, before adsorption, that the construction of HPCG showed a porous net structure [Fig. 5(D)], which was similar to the previous TAMAC homo-polymer gel [Fig. 2(A)]. After HPCG adsorption, the porous structure of HPCG disappeared [Fig. 5(E)], possibly because the formed insoluble flocs (derived from the interactions between the soluble plane-like polycations in the HPCG system and the anionic dyes) could be transferred to the HPCG gel-skeletons (*i.e.* the solid insoluble network constructions in the HPCG system), to fill the porous spaces of HPCG, so as to achieve the super-high adsorption capacity of the HPCG product. Thus, the results of SEM analysis for HPCG adsorptions were in accordance with those of optical microscopy analysis, further confirming that the obtained HPCG adsorbents would show the intelligent adsorption behaviours.

The XRD spectrum of the HPCG product before adsorption in Fig. 6(A) showed a lot of sharp peaks for the crystalline phases (attributed to the soluble plane-like polycations in the HPCG system), besides a halo peak for the amorphous phase (attributed to the insoluble network gel-skeletons in the HPCG system), further confirming that the HPCG products had two phase structures (*i.e.* the heterogeneous structures), which were consistent with the results in the previous Section 3.2. After adsorption, the sharp peaks for the crystalline phases were absent from the XRD spectrum of the HPCG product [Fig. 6(B)*versus*Fig. 6(A)], possibly because the soluble plane-like polycations (with the crystalline structures) in the HPCG system could be interacting with the anionic dyes to form the amorphous combinations (*i.e.* the formed flocs), besides that the insoluble network gel-skeletons in the HPCG system could adsorb the anionic dyes, which was one part of the intelligent adsorption behaviours of the HPCG system.

In addition, the XPS further showed, compared to the analysis of the HPCG product before adsorbing the dyes [Fig. 7(A–C)], that after adsorbing the coloured dyes of Reactive Scarlet 3BS [Fig. 7(D–F)], the binding energy of N1s of HPCG product was increased from 401.79 eV to 402.08 eV, possibly because the N-containing cation of HPCG product had formed the strong binding interactions with the anionic dyes after adsorption. However, the binding energies of other elements [*e.g.* C1s, Fig. 7(C)*versus*Fig. 7(F)] before and after HPCG adsorption changed a little, which could suggest that the general combination states of the homologous–heterogeneous components in the HPCG system after adsorption were the same as those before adsorption. Before adsorption, the soluble plane-like polycations could be mixed and interacted evenly with the insoluble network gel-skeletons in the HPCG system. When the obtained HPCG products interacted with the anionic dyes in water, the soluble plane-like polycations would be freely delivered from HPCG system to water phase, to form the insoluble flocs with the anionic dyes. If the combination states of the homologous–heterogeneous components (*i.e.* the soluble plane-like polycations and the insoluble network gel-skeletons) in the HPCG system before and after adsorption were the same, this would mean that the formed insoluble flocs (derived from the interactions between the soluble plane-like polycations in the HPCG system and the anionic dyes) should be re-transferred to the HPCG gel-skeletons at the later stage of HPCG adsorption, so as to recover the combination states before adsorption.

The information on homologous–heterogeneous structure transformations mentioned above lead us to confirm that the obtained HPCG products show the intelligent adsorption behaviours: first, the HPCG gel-skeletons could absorb the anionic dyes and the soluble plane-like polycations would be freely delivered from the HPCG system to form the insoluble flocs with the anionic dyes in water. Subsequently, the formed flocs re-transferred to the solid gel skeletons, so that the anionic dyes could be efficiently removed from water phase.

### 3.6 Adsorption models for HPCG adsorption

Several typical adsorption isotherm models and adsorption kinetics models were selected to further investigate the adsorption behaviours of HPCG.

**Fig. 8:**
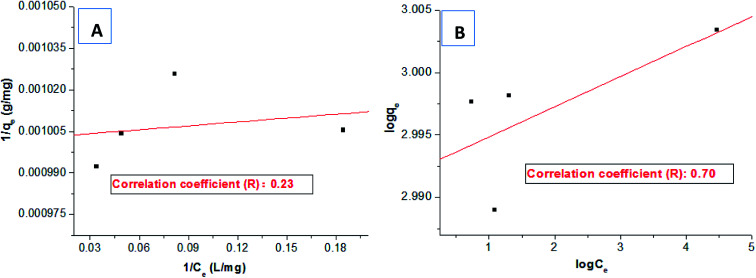
Fitting the Langmuir model (A) and Freundlich model (B) using adsorption data.

Firstly, based on the dye removal percentages at different dosages in Fig. 3, the equilibrium dye concentration in the solution (*C*_e_) and the equilibrium adsorption capacity (*q*_e_) of HPCG could be further calculated. The adsorption data at the saturated adsorption states with the dosages of 0.007–0.01 g were selected to fit the typical single molecular layer adsorption models (*i.e.* Langmuir and Freundlich models) for evaluating the adsorption isotherm on the variable relationships between *q*_e_ and *C*_e_. The results are shown in Fig. 8. The results show that the equilibrium data did not fit the Langmuir model [Fig. 8(A)] or Freundlich model [Fig. 8(B)] well, and the correlation coefficients (*R*) were only 0.23 and 0.70, respectively, indicating that HPCG adsorption did not follow a typical single molecular layer adsorption process.

**Fig. 9:**
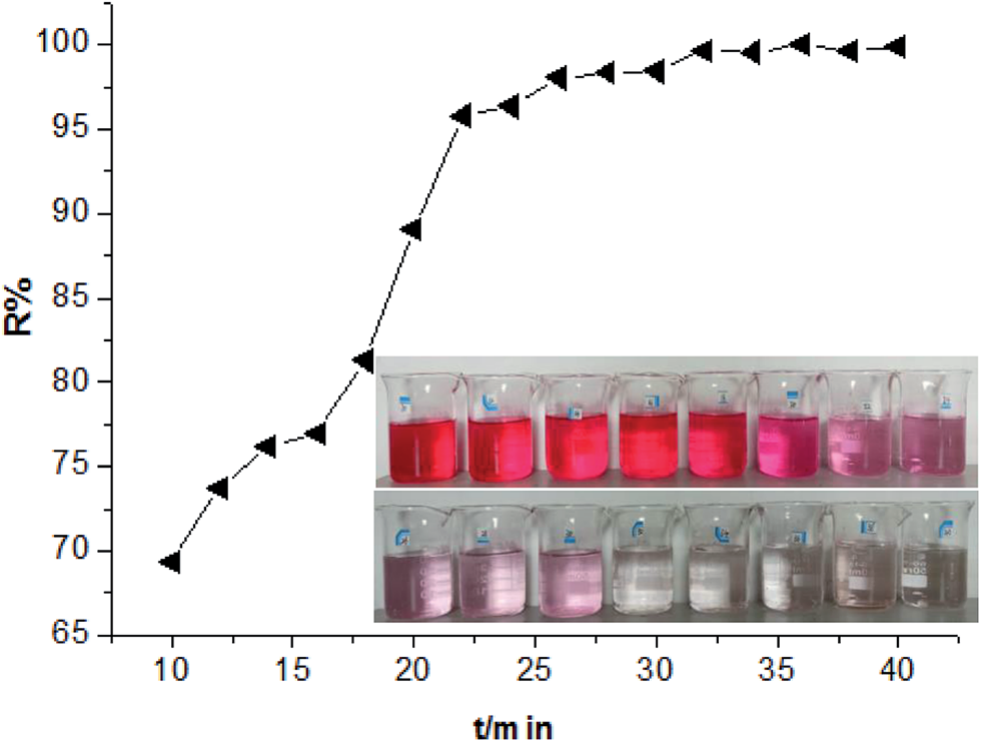
Comparing the adsorption results of HPCG [*n*_(TAMAC)_/*n*_(TADMAC)_ = 60/40] at different adsorption time (10–40 min).

Subsequently, in order to further investigate the adsorption behaviours of HPCG, the adsorption kinetics of HPCG were also studied. According to the experimental procedure in Section 2.3, experiments on the adsorption of HPCG [*n*_(TAMAC)_/*n*_(TADMAC)_ = 60/40] at different adsorption time (10–40 min) were carried out and the results shown in Fig. 9. It took 32 min for 0.016 g of HPCGs to remove 99.68% of the dyes in water, and the dye removal percentages changed very little after 32 min, indicating that adsorption equilibrium was reached at the 32^nd^ minute.

**Fig. 10:**
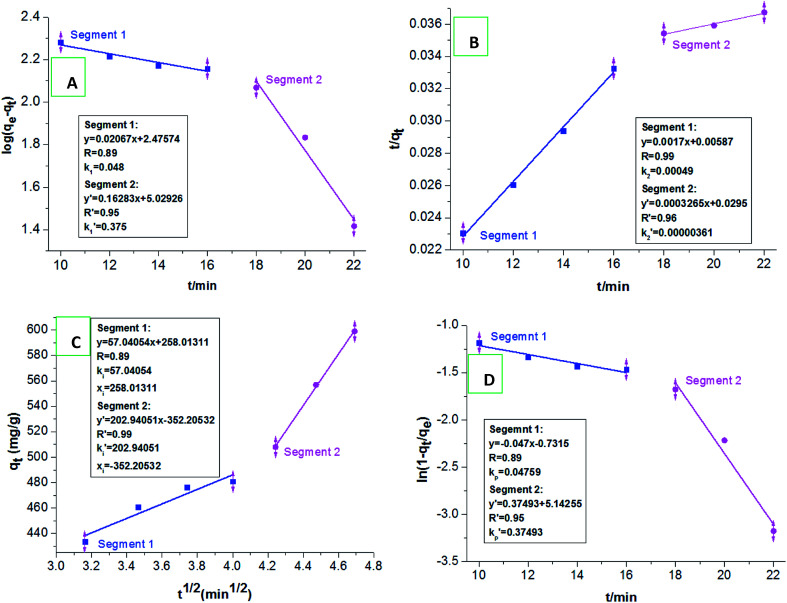
Fitting the pseudo-first order kinetics (A), pseudo-second order kinetics (B), intraparticle diffusion (C), and particle diffusion equations (D) in two segments.

We selected the adsorption data before achieving the adsorption equilibrium (10–22 min), to fit the pseudo-first order kinetics, pseudo-second order kinetics, intraparticle diffusion, and particle diffusion models; and conducted a comparative study of the effect on these models on dye adsorption. We discovered that the distributions of the selected adsorption data points in all the adsorption kinetics models were not in the same straight line, indicating that the total adsorption process of HPCG could not be directly explained by linear plots of the selected adsorption kinetics models. For this, we further fitted the adsorption data into two segments (Segment 1: the adsorption data points at 10–16 min, Segment 2: the adsorption data points at 18–22 min) in the adsorption kinetics models, and the results are shown in Fig. 10(A–D). The results show that the adsorption data could well follow the adsorption kinetics models (*i.e.* pseudo-first order kinetic, pseudo-second order kinetic, intra-particle diffusion, and particle diffusion models) with high correlation coefficients (*R* or *R*′) of ≥ 0.89, when they are fitted into two segments, indicating that the adsorptions of HPCG could be carried out in two segments. For the overall comparison of the corresponding kinetic parameters in Segment 1 and Segment 2, all the adsorption rate constants corresponding to the pseudo-first order kinetic [Fig. 10(A)], intra-particle diffusion [Fig. 10(C)], and particle diffusion models [Fig. 10(D)], were significantly higher than those corresponding to the pseudo-second order kinetic [Fig. 10(B)] in Segment 1 (*k*_2_ = 4.9 × 10^−4^ g mg^−1^ min^−1^) and Segment 2 
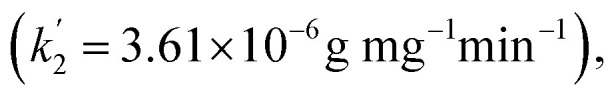
 indicating that the total adsorption process of HPCG mainly follows the pseudo-first order kinetic, intra-particle diffusion, and particle diffusion models. For a more detailed comparison, the adsorption rate constants in Segment 1 corresponding to the pseudo-first order kinetic (*k*_1_ = 0.048 min^−1^), intra-particle diffusion (*k*_i_ = 57.041 mg g^−1^ min^1/2^), and particle diffusion models (*k*_p_ = 0.048 min^−1^) were far lower than those in Segment 2 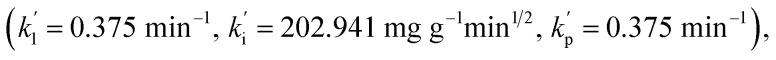 indicating that the HPCG adsorption in Segment 1 was a relatively slow process likely to be the rate control segment, and Segment 2 was a suddenly changing process likely to be the acceleration segment. In Segment 1, the HPCG product was firstly swelling to keep the solid forms of the gel skeletons in water and deliver the soluble polycations into water phase as the liquid forms of the anionic-dye scavengers. Subsequently, whilst the solid gel skeletons could provide more spaces to absorb and accommodate the anionic dyes, the soluble polycations delivered into water could also catch the anionic dyes, to form the insoluble flocs with the anionic dyes and separate them from the water phase. Thus, it took a relatively long time to complete the complicated interaction processes mentioned above in Segment 1. In Segment 2, the formed flocs could be transferred to the solid gel skeletons to be further fixed due to the associations between the alkyl groups in the gel skeletons and those in the formed flocs, which had been similarly proven in our previous work to be a very fast process,^32^ thus resulting in a suddenly changing process in this stage.

Generally, as known from the above-mentioned results on the HPCG adsorption models, the HPCG adsorption follows the new two-segment adsorption process, *i.e.* including a rate control segment and an acceleration segment, to further confirm the existence of the intelligent adsorption behaviours, which agree with the previous results on the homologous–heterogeneous structure transformations during HPCG adsorption.

## 4. Conclusion

A homologous–heterogeneous polycationic gel (HPCG) system was constructed by a waste-free synthesis process, so that the HPCG product could be directly used as a super-efficient adsorbent material for purifying dyeing wastewater without any treatments and waste. It is the first discovery of a new intelligent adsorption effect occurring in HPCG adsorption by detecting the homologous–heterogeneous structure transformations in HPCG adsorption using optical microscopy, SEM, XRD, and XPS analysis technologies.

Under the same application conditions, the adsorption capacities of HPCG products were 532.55–605.45 times and 3.67–46.05 times higher than that of the widely-used activated carbon and those of the similar polycationic cotton adsorbents reported in our previous serial works, respectively, which was regarded as the greatest improvement on the adsorption ability of HPCG *versus* this existing adsorbent and demonstrated more efficient purification of dyeing wastewater than we could do before.

In addition, through studying the adsorption models, it was further discovered that HPCG adsorption followed a new two-segment adsorption process, consisting of a speed control segment and an acceleration segment, also confirming the existence of the intelligent adsorption effect for HPCG adsorption. The intelligent adsorption effect of HPCG adsorption could be explained as follows: the insoluble network polycations in the HPCG system were swelling as the solid forms of the gel skeletons, providing more spaces to absorb and accommodate the anionic dyes. Meanwhile, the soluble plane-like polycations in the HPCG system were freely delivered into water phase, to efficiently catch the anionic dyes, forming the insoluble flocs with the anionic dyes, and then the formed flocs were transferred to the solid gel skeletons to be further fixed and be separated from the water phase.

## Conflicts of interest

There are no conflicts to declare.

## Supplementary Material
